# Comparing 2 National Organization-Level Workplace Health Promotion and Improvement Tools, 2013–2015

**DOI:** 10.5888/pcd13.160164

**Published:** 2016-09-29

**Authors:** Amy Meador, Jason E. Lang, Whitney D. Davis, Nkenge H. Jones-Jack, Qaiser Mukhtar, Hua Lu, Sushama D. Acharya, Meg E. Molloy

**Affiliations:** Author Affiliations: Jason E. Lang, Qaiser Mukhtar, Hua Lu, National Center for Chronic Disease Prevention and Health Promotion, Centers for Disease Control and Prevention, Atlanta, Georgia; Whitney D. Davis, Meg E. Molloy, Prevention Partners, Chapel Hill, North Carolina; Nkenge H. Jones-Jack, Carter Consulting, Inc, Atlanta, Georgia; Sushama D. Acharya, CDC Foundation, Atlanta, Georgia.

## Abstract

Creating healthy workplaces is becoming more common. Half of employers that have more than 50 employees offer some type of workplace health promotion program. Few employers implement comprehensive evidence-based interventions that reach all employees and achieve desired health and cost outcomes. A few organization-level assessment and benchmarking tools have emerged to help employers evaluate the comprehensiveness and rigor of their health promotion offerings. Even fewer tools exist that combine assessment with technical assistance and guidance to implement evidence-based practices. Our descriptive analysis compares 2 such tools, the Centers for Disease Control and Prevention’s Worksite Health ScoreCard and Prevention Partners’ WorkHealthy America, and presents data from both to describe workplace health promotion practices across the United States. These tools are reaching employers of all types (N = 1,797), and many employers are using a comprehensive approach (85% of those using WorkHealthy America and 45% of those using the ScoreCard), increasing program effectiveness and impact.

## Introduction

Each year, preventable chronic diseases, such as heart disease, stroke, cancer, and diabetes, cause 70% of all deaths in the United States and account for 7 of the top 10 causes of death (1,2). Individuals with 1 or more chronic conditions accounted for 86% of all US health care spending in 2010 (3). Given that employed individuals spend half of their waking hours at work, workplaces present an opportunity to influence and improve individual health behaviors (4). Noted long-term benefits of comprehensive workplace health promotion interventions include improved health outcomes, reduced absenteeism, improved employee morale, higher employee retention, and reduced health care costs (5–8).

Interventions to create healthy workplaces are becoming more common; half of employers with more than 50 employees offer some type of health promotion program (9). However, few employers implement a comprehensive approach using evidence-based interventions that achieve the health and cost improvements employers seek (10,11). A 2004 national worksite study found that only 6.9% of employers offer comprehensive programs as defined by Healthy People 2010 (12). Comprehensive interventions influence health at the individual, interpersonal, organizational, and environmental levels (13,14). Such interventions ensure workplace policies, benefits, built environment, programs, and evaluation work together in synergistic ways to create healthy workplaces. For example, providing information to employees about the importance of physical activity is more effective when there are appropriate facilities, time, and opportunities to be physically active during the workday (15).

A small number of organization-level assessment and benchmarking tools have emerged to help employers evaluate the comprehensiveness and effectiveness of their healthy workplace practices. Even fewer tools combine comprehensive assessments with technical support for implementing evidence-based practices. We examined the similarities and differences of 2 such tools, the CDC Worksite Health ScoreCard and Prevention Partners’ WorkHealthy America, including an analysis of combined data to describe workplace health promotion practices across the United States.

## Tools Studied

### CDC Worksite Health ScoreCard

**Figure 1:**
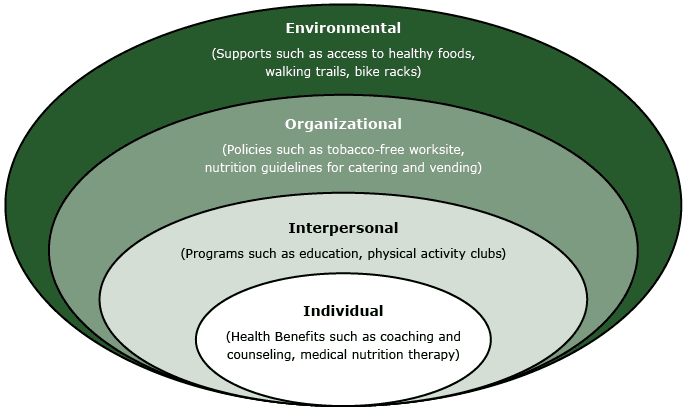
The levels of influence from the socio-ecological model with examples of intervention strategies recommended in Prevention Partners’ WorkHealthy America and the CDC Worksite Health ScoreCard.

The CDC Worksite Health ScoreCard is an organization-level self-assessment and evaluation tool for employers to determine the number of evidence-based health promotion interventions in their worksites that prevent chronic diseases (16). The ScoreCard consists of 125 yes/no questions organized across 16 health domains and topics, including lifestyle risk factors such as physical inactivity and poor diet, disease conditions such as high blood pressure and diabetes, and wellness program infrastructure. The questions reflect 4 main intervention types — programs, policies, environmental supports, and benefits — that align with the socio-ecological model’s levels of influence on individual behavior (Figure 1).

Each item in the ScoreCard has a score of 0, 1, 2, or 3, with scores determined by the strength of scientific evidence for that strategy and the health impact on the workforce population (17); the higher the score, the stronger the evidence and the larger the impact. A 0 score is given when an employer does not have a recommended strategy in place. All interventions in the ScoreCard are evidence-based.

CDC began development of the ScoreCard in 2008 in collaboration with academic, governmental, and private sector experts. First, existing workplace health tools and reliable, valid questions from other instruments were examined (18). New topics and questions were then developed to address gaps identified through literature reviews and surveys of state health departments. Twelve original topics were pretested with a small number of employers to ensure comprehension. After revisions to incorporate employer feedback, the ScoreCard was fully field tested with 93 employers to determine content and face validity, inter-rater reliability, and feasibility of adoption (19). The instrument was then finalized, and a hard-copy version was released in 2012. The ScoreCard was updated in 2013 to include 4 additional topics, including lactation supports, occupational health and safety, vaccine-preventable diseases, and community resources, which were tested by using similar methods.

In 2014, the ScoreCard was released as a free, online application allowing employers to track their workplace health improvements over time, benchmark their scores against other users, and prioritize feasible strategies to strengthen employers’ wellness investments. The online system enables CDC to aggregate data, identify gaps, monitor trends, and develop additional tools to support healthy workplaces.

### Prevention Partners’ WorkHealthy America

WorkHealthy America is a web-based assessment, benchmarking, and strategic planning tool for employers focused on nutrition, physical activity, tobacco use cessation, and culture of wellness. The 125 indicators are aligned with the socio-ecological model to measure policies, benefits, and environments that affect employee health (Figure 1). WorkHealthy America provides automated, tailored recommendations reports and action plans to help workplace leadership implement improvements. An executive summary benchmarks current practices against national standards and peer organizations. Workplaces also have access to searchable online toolboxes and coaching support.

Every question in WorkHealthy America is weighted according to the strength of evidence, with 3 points for strongly evidence-based concepts, 2 points for key process measures with less evidence, and 1 point for promising practices. Additional questions provide information on how practices are implemented and are not scored. The total score is translated into a letter grade using a standardized grading scale.

Prevention Partners developed WorkHealthy America based on 15 years of experience strengthening chronic disease prevention activities in the private sector. This experience, combined with a thorough literature review, provided the groundwork for the assessment. Prevention Partners then drafted a conceptual model and questions for each topic and solicited feedback from national experts. Questions were pilot tested with employers for additional content and face validation. In 2008, WorkHealthy America launched with modules on nutrition, physical activity, and tobacco. A culture of wellness module was added in 2012. Modules are periodically revised to ensure indicators and weighting are current with the latest evidence.

## Comparison of the Tools

The content and recommended strategies covered by each tool are strikingly similar. Both focus on actions employers, rather than employees, can take to create a culture that supports health; and both include strategies at all levels of the socio-ecological model. Although the instruments were developed independently, the processes were similar. Each organization conducted extensive reviews of workplace health literature and consulted with national subject matter experts. Many of the same sources of evidence were used to determine the final composition of the instruments, such as the CDC Guide to Community Preventive Services. Each organization arrived at similar interpretations of the evidence base, demonstrated in 88% agreement in concepts covered and scoring among questions common to both instruments. Both are of similar length and address the topics of physical activity, nutrition, tobacco use, and organizational infrastructure strategies. Each organization agrees these “core 4” topics are paramount for healthy workplaces and have an impact on many related health risks and conditions. Both instruments have undergone some validation (19).

Several differences exist between the tools. In addition to the core 4 topics, the ScoreCard asks about leading chronic conditions such as diabetes and cardiovascular disease, occupational safety and health, and vaccine-preventable diseases. This approach enables the ScoreCard to cover more issues but limits the number of questions for each. In contrast, WorkHealthy America allows for more depth within topics. WorkHealthy America also provides tailored recommendations and action plans to guide employers to improve their practices.

Another administrative difference is the flexibility given to employers when completing the instruments. WorkHealthy America does not restrict the frequency of assessment submissions, and users may complete 1 module at a time. This approach was chosen so that worksites could elect to focus their improvement efforts on topics of their choice, although 71% (n = 476) have completed assessments in all 4 modules. In contrast, the ScoreCard requires users to answer all questions in all modules to generate reports. With this approach, the ScoreCard provides employers more complete benchmarking data. The ScoreCard restricts online submission to no more than annually, but a paper-and-pencil version allows employers to assess more frequently.

## How Communities Are Using the Tools

The ScoreCard was an important component of CDC efforts to build comprehensive employer wellness programs through 2 national demonstration programs showcased in a series of employer case studies. State health departments and their partners have used the ScoreCard to expand implementation of evidence-based worksite practices. Idaho has used the ScoreCard since 2013, funding 7 local public health districts to each assist 10 employers in creating sustainable workplace wellness programs. Since the initiative began, approximately 90 employers have used the ScoreCard. Similarly, South Dakota requires partners to use the ScoreCard to measure how they are implementing the 2008 physical activity guidelines in worksites. North Carolina is using the ScoreCard to guide and evaluate worksites as part of a broader CDC-funded state-based program focused on environmental strategies that support healthful behaviors to address obesity. The state health department is supporting regional worksite coordinators to improve employer programs and track select practices.

WorkHealthy America has been disseminated through strategic alliances with statewide, sector-specific, and county-level initiatives (http://forprevention.org/p2/prevention-stories/). The Healthy NC Hospital Initiative used WorkHealthy America to support all 136 North Carolina hospitals to voluntarily adopt tobacco-free policies, establish healthy food environments, and increase opportunities for physical activity at work. Building on that work, Healthy Together North Carolina is a multisector collaborative using WorkHealthy America to support at least 10 of the largest employers in every North Carolina county to become healthy workplaces by 2025. Other initiatives using WorkHealthy America include hospital association or health department-led healthy workplace collaborations in South Carolina, Oklahoma, Virginia, and New York City.

## Analysis of Combined Data

**Figure 2:**
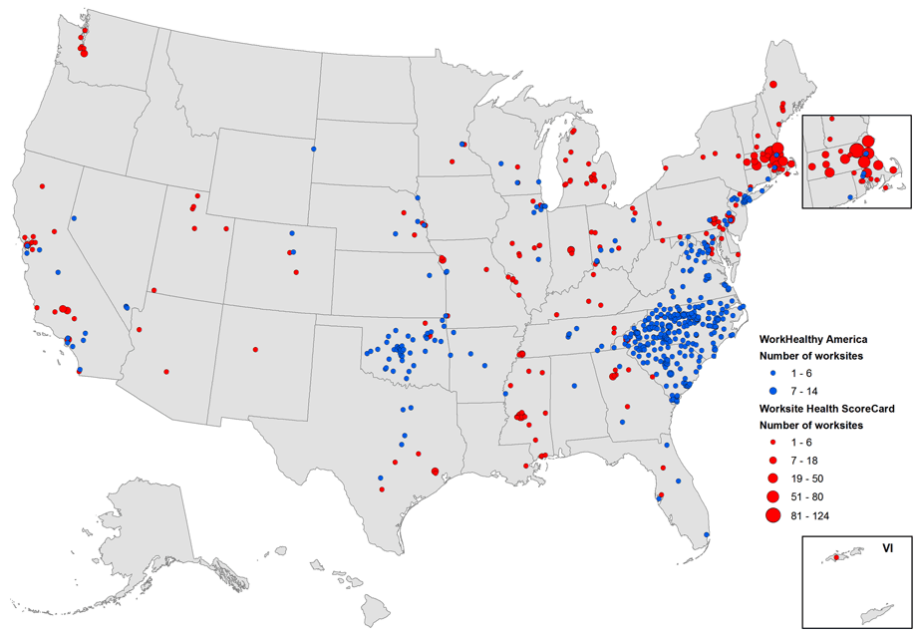
Workplaces using WorkHealthy America (2008–2015, N = 667) and the CDC Worksite Health ScoreCard (2014–2015, N = 1,124). Workplaces were mapped to city, county, or zip code depending on availability of geographic data. Excluded are 6 workplaces because of lack of geographic information. Abbreviation: VI, Virgin Islands.

Since their launch, the ScoreCard and WorkHealthy America together have reached 1,797 workplaces across 42 states; the workplaces are diverse in size, sector, and location (Table 1). Most ScoreCard users (83%) are small employers (≤249 employees), with the largest sectors representing health care and social assistance (11%). Most WorkHealthy America users are hospitals (44%), followed by manufacturing (14%) and local government (13%). ScoreCard data were geocoded to city or county centroid, and WorkHealthy America data were geocoded to zip code centroid by using ArcGIS 10.3.1 (Esri) (Figure 2).

**Table 1:** Overview of Prevention Partners’ WorkHealthy America and CDC Worksite Health ScoreCard Tools, Data Through September 30, 2015

Demographics	Prevention Partners WorkHealthy America	CDC Worksite Health ScoreCard
**Total number of organizations**	667	1,064
Organizations having taken multiple assessments, n (%)	380 (57)	68 (6)
**Size of employer, n (%)**		
1–99 employees	97 (15)	813 (72)
100–249 employees	101 (15)	122 (11)
250–749 employees	175 (26)	95 (8)
≥750 employees	237 (36)	100 (9)
Unknown	57 (9)	0
**Worksite structure, n (%)**		
For-profit	189 (28)	873 (78)
Nonprofit	223 (33)	98 (9)
Government	190 (29)	153 (14)
Unknown	65 (10)	0
**Number of states represented**	31	36
**About the assessment**		
Initial release	2008	2012
Last updated	2014	2014
Unit of analysis	Worksite	Worksite
Length	125 questions	125 questions
Topics/modules	4	16
Question type	Yes/no and multiple choice	Yes/no
Validated	Yes	Yes
Cost	Grant funded and/or licensing fee (4 tiers)	No
**Data collection**		
Self-report	Yes, plus organizational attestation, and confirmation by Prevention Partners for organizations seeking recognition	Yes
Administration	Online	Paper-and-pencil or online
Recommended frequency of assessment	Two times/y, but available any time	Annually
Representative sample	No	No
Scoring	Points based (weighted) and letter grade	Points based (weighted)
**Technical assistance**		
Tailored benchmarking report	Yes	Yes
Recommendations provided	Yes	No
Action plans provided	Yes	In development
Access to tools and resources	Yes	Yes
Additional support	Recognition for high performance, public mapping of participation and recognition, online user guide, group trainings, telephone-based coaching, webinar series, e-newsletters, video success stories, in-person trainings, some onsite review and consultation	Video tutorials, online user guide, telephone-based coaching, webinars

A subset of 26 common indicators that had high levels of agreement in both question content and scoring were identified for analysis. Selected questions were categorized by health topic: organizational supports and culture of wellness (n = 7), tobacco control (n = 7), nutrition (n = 4), lactation support (n = 3), and physical activity (n = 5). In addition, questions were classified in 1 of 5 intervention types (leadership support, program, policy, benefits, or environmental supports).

Employers that used either instrument between September 30, 2013, and September 30, 2015, were included in the analysis. Data were self-reported by employers and collected through the online assessment feature in each tool. The number of employers using WorkHealthy America (n < 300) varied by health topic because employers are not required to complete every module. The number of employers using the ScoreCard (n = 1,130) was consistent across topics. Both surveys include skip patterns that influence the number of responses to indicators. Table 2 provides the side-by-side comparison of the 26 selected indicators.

**Table 2:** Selected Common Indicators in Prevention Partners’ WorkHealthy America and CDC Worksite Health ScoreCard Tools, by Size of Employer, September 30, 2013, Through September 30, 2015

Indicator (Intervention Type)	Prevention Partners WorkHealthy America		CDC Worksite Health ScoreCard	
	No. of Employers Using the Survey	Employers Implementing the Strategy	No. of Employers Using the Survey	Employers Implementing the Strategy
		n (%)		n (%)
**Organizational Support and Culture of Wellness**				
**Health risk appraisal with individual feedback (leadership)**	**275**	**201 (73)**	**1,130**	**476 (42)**
1–99 employees	43	36 (84)	813	285 (35)
100–249 employees	40	31 (78)	122	52 (43)
250–749 employees	69	48 (70)	95	60 (63)
≥750 employees	115	83 (72)	100	79 (79)
Unknown size	8	3 (38)	0	
**Organizational commitment to wellness (leadership)**	**275**	**242 (88)**	**1,130**	**571 (51)**
1–99 employees	43	38 (88)	813	382 (47)
100–249 employees	40	38 (95)	122	70 (57)
250–749 employees	69	61 (88)	95	56 (59)
≥750 employees	115	101 (88)	100	63 (63)
Unknown size	8	4 (50)	0	
**Use of incentives or disincentives to increase participation (leadership)**	**307**	**281 (92)**	**1,130**	**553 (49)**
1–99 employees	45	41 (91)	813	341 (42)
100–249 employees	46	39 (85)	122	63 (52)
250–749 employees	79	74 (94)	95	67 (71)
≥750 employees	128	119 (93)	100	82 (82)
Unknown size	9	8 (89)	0	
**Wellness committee (leadership)**	**275**	**218 (79)**	**1,130**	**456 (40)**
1–99 employees	43	36 (84)	813	252 (31)
100–249 employees	40	27 (68)	122	65 (53)
250–749 employees	69	53 (77)	95	61 (64)
≥750 employees	115	97 (84)	100	78 (78)
Unknown size	8	5 (63)	0	
**Paid staff for health promotion (leadership)**	**275**	**228 (83)**	**1,130**	**287 (25)**
1–99 employees	43	35 (81)	813	138 (17)
100–249 employees	40	28 (70)	122	37 (30)
250–749 employees	69	55 (80)	95	39 (41)
≥750 employees	115	106 (92)	100	73 (73)
Unknown size	8	4 (50)	0	
**Annual budget for health promotion (leadership)**	**275**	**220 (80)**	**1,130**	**396 (35)**
1–99 employees	43	34 (79)	813	236 (29)
100–249 employees	40	31 (78)	122	41 (34)
250–749 employees	69	57 (83)	95	48 (51)
≥750 employees	115	94 (82)	100	71 (71)
Unknown size	8	4 (50)	0	
**Clearly stated wellness goals (leadership)**	**275**	**185 (67)**	**1,130**	**347 (31)**
1–99 employees	43	26 (60)	813	203 (25)
100–249 employees	40	24 (60)	122	41 (34)
250–749 employees	69	50 (72)	95	41 (43)
≥750 employees	115	83 (72)	100	62 (62)
Unknown size	8	2 (25)	0	
**Tobacco Control**				
**FDA-approved cessation medications at no or low cost^a^ (benefits)**	**288**	**257 (89)**	**1,130**	**728 (64)**
1–99 employees	45	42 (93)	813	488 (60)
100–249 employees	45	40 (89)	122	87 (71)
250–749 employees	74	67 (91)	95	67 (71)
≥750 employees	117	105 (90)	100	86 (86)
Unknown size	7	3 (43)	0	
**Nicotine replacement therapy at no or low cost (benefits)**	**288**	**232 (81)**	**1,130**	**594 (53)**
1–99 employees	45	39 (87)	813	403 (50)
100–249 employees	45	34 (76)	122	60 (49)
250–749 employees	74	63 (85)	95	57 (60)
≥750 employees	117	92 (79)	100	74 (74)
Unknown size	7	4 (57)	0	
**Signage about tobacco-free policy (environmental supports)**	**288**	**204 (71)**	**1,130**	**649 (57)**
1–99 employees	45	33 (73)	813	444 (55)
100–249 employees	45	27 (60)	122	64 (52)
250–749 employees	74	53 (72)	95	63 (66)
≥750 employees	117	88 (75)	100	78 (78)
Unknown size	7	3 (43)	0	
**Written policy banning tobacco use (policy)**	**288**	**219 (76)**	**1,130**	**749 (66)**
1–99 employees	45	33 (73)	813	520 (64)
100–249 employees	45	31 (69)	122	87 (71)
250–749 employees	74	54 (73)	95	59 (62)
≥750 employees	117	97 (83)	100	83 (83)
Unknown size	7	4 (57)	0	
**Active enforcement of tobacco-free policy (policy)**	**288**	**223 (77)**	**1,130**	**673 (60)**
1–99 employees	45	30 (67)	813	467 (57)
100–249 employees	45	33 (73)	122	79 (65)
250–749 employees	74	53 (72)	95	54 (57)
≥750 employees	117	103 (88)	100	73 (73)
Unknown size	7	4 (57)	0	
**Refer tobacco users to quitline or other services (program)**	**288**	**206 (72)**	**1,130**	**505 (45)**
1–99 employees	45	23 (51)	813	301 (37)
100–249 employees	45	25 (56)	122	59 (48)
250–749 employees	74	53 (72)	95	61 (64)
≥750 employees	117	101 (86)	100	84 (84)
Unknown size	7	4 (57)	0	
**Provide free or subsidized tobacco use cessation counseling (program)**	**288**	**245 (85)**	**1,130**	**569 (50)**
1–99 employees	45	37 (82)	813	333 (41)
100–249 employees	45	36 (80)	122	73 (60)
250–749 employees	74	64 (86)	95	70 (74)
≥750 employees	117	105 (90)	100	93 (93)
Unknown size	7	3 (43)	0	
**Nutrition**				
**Label foods with nutritional information^b^ (environmental supports)**	**290**	**160 (55)**	**801^c^**	**204 (26)**
1–99 employees	43	7 (16)	512	120 (23)
100–249 employees	44	19 (43)	104	18 (17)
250–749 employees	77	39 (51)	88	26 (30)
≥750 employees	118	92 (78)	97	40 (41)
Unknown size	8	3 (38)	0	
**Identify healthier food and beverages with sign or symbol (environmental supports)**	**290**	**161 (56)**	**801^c^**	**266 (33)**
1–99 employees	43	14 (33)	512	138 (27)
100–249 employees	44	18 (41)	104	36 (35)
250–749 employees	77	41 (53)	88	42 (48)
≥750 employees	118	84 (71)	97	50 (52)
Unknown size	8	4 (50)	0	
**Use pricing to encourage purchase of healthy options^b^ (policy)**	**290**	**135 (47)**	**801^c^**	**112 (14)**
1–99 employees	43	15 (35)	512	56 (11)
100–249 employees	44	14 (32)	104	20 (19)
250–749 employees	77	37 (48)	88	18 (20)
≥750 employees	118	64 (54)	97	18 (19)
Unknown size	8	5 (63)	0	
**Provide free or subsidized nutrition counseling or self-management programs on healthy eating (program)**	**290**	**271 (93)**	**1,130**	**364 (32)**
1–99 employees	43	39 (91)	813	203 (25)
100–249 employees	44	41 (93)	122	41 (34)
250–749 employees	77	70 (91)	95	45 (47)
≥750 employees	118	113 (96)	100	75 (75)
Unknown size	8	8 (100)	0	
**Lactation Support**				
**Private area to breastfeed (environmental supports)**	**290**	**211 (73)**	**1,117^d^**	**720 (64)**
1–99 employees	43	32 (74)	813	493 (61)
100–249 employees	44	24 (55)	122	88 (72)
250–749 employees	77	56 (73)	95	68 (72)
≥750 employees	118	93 (79)	87	71 (82)
Unknown size	8	6 (75)	0	
**Flexible time for breastfeeding (policy)**	**290**	**197 (68)**	**1,117^d^**	**766 (69)**
1–99 employees	43	30 (70)	813	522 (64)
100–249 employees	44	23 (52)	122	94 (77)
250–749 employees	77	50 (65)	95	73 (77)
≥750 employees	118	88 (75)	87	77 (89)
Unknown size	8	6 (75)	0	
**Paid maternity leave (policy)**	**290**	**129 (44)**	**1,117^d^**	**393 (35)**
1–99 employees	43	26 (60)	813	285 (35)
100–249 employees	44	22 (50)	122	38 (31)
250–749 employees	77	39 (51)	95	34 (36)
≥750 employees	118	40 (34)	87	36 (41)
Unknown size	8	2 (25)	0	
**Physical Activity**				
**Access to exercise facilities on site (environmental supports)**	**214**	**181 (85)**	**1,130**	**330 (29)**
1–99 employees	43	28 (65)	813	203 (25)
100–249 employees	29	23 (79)	122	31 (25)
250–749 employees	57	50 (88)	95	32 (34)
≥750 employees	81	77 (95)	100	64 (64)
Unknown size	4	3 (75)	0	
**Environmental supports for physical activity (environmental supports)**	**280**	**251 (90)**	**1,130**	**513 (45)**
1–99 employees	45	39 (87)	813	301 (37)
100–249 employees	41	36 (88)	122	59 (48)
250–749 employees	74	63 (85)	95	67 (71)
≥750 employees	112	105 (94)	100	86 (86)
Unknown size	8	8 (100)	0	
**Use point-of-decision prompts to encourage physical activity^b^ (environmental supports)**	**280**	**148 (53)**	**1,130**	**170 (15)**
1–99 employees	45	22 (49)	813	98 (12)
100–249 employees	41	15 (37)	122	17 (14)
250–749 employees	74	35 (47)	95	20 (21)
≥750 employees	112	73 (65)	100	35 (35)
Unknown size	8	3 (38)	0	
**Discount for local or onsite exercise facility (policy)**	**280**	**228 (81)**	**1,130**	**578 (51)**
1–99 employees	45	26 (58)	813	382 (47)
100–249 employees	41	24 (59)	122	57 (47)
250–749 employees	74	66 (89)	95	61 (64)
≥750 employees	112	105 (94)	100	78 (78)
Unknown size	8	7 (88)	0	
**Organized programs or peer support for physical activity (program)**	**214**	**186 (87)**	**1,130**	**446 (39)**
1–99 employees	43	38 (88)	813	273 (34)
100–249 employees	29	26 (90)	122	47 (39)
250–749 employees	57	47 (82)	95	48 (50)
≥750 employees	81	74 (91)	100	78 (78)
Unknown size	4	1 (25)	0	

Abbreviation: FDA, Food and Drug Administration.

a Intervention was in the top quartile for both surveys.

b Intervention was in the bottom quartile for both surveys.

c This question did not apply to 329 employers that did not provide food or beverages at the worksite, so this question was skipped.

d Thirteen employers completed ScoreCard in 2013 before the release of the breastfeeding module.

The number of employers answering that that they had implemented the selected strategies varied between the 2 instruments. In general, employers using WorkHealthy America showed a higher proportion of recommended practices in place compared with those using the ScoreCard. Prevalence estimates across WorkHealthy America strategies ranged from 44% to 93%; and prevalence estimates of ScoreCard strategies ranged from 14% to 69%. There are several possible explanations for these differences. The ScoreCard data primarily represents baseline assessments, whereas most WorkHealthy America users have reassessed at least once. Among organizations that reassessed (N = 380 for WorkHealthy America and N = 68 for the ScoreCard), the average improvement per indicator was 3.4 percentage points for WorkHealthy America (range, 1 to 9 percentage points, with the greatest improvement in organizations using point-of-decision prompts for physical activity) and 27.7 percentage points for the ScoreCard (range, 10.3 to 51.8 percentage points, with the greatest improvement in organizations providing health risk appraisals with feedback). WorkHealthy America has been available for a longer period of time, giving employers more time to improve. Employers using WorkHealthy America receive tailored technical assistance, and Prevention Partners’ leadership engagement model with community, corporate, and statewide partnerships increases readiness and uptake of practices among employers. Lastly, one-third of WorkHealthy America users are large employers (≥750 employees), and most users are hospitals, which could be a higher-performing sector. ScoreCard users are more frequently small or midsized (≤249 employees) employers. Analysis of the indicators stratified by size confirmed that, for most indicators, the largest workplaces (≥750 employees) performed the best (Table 2). This aligns with previous studies showing that smaller employers face different challenges in resources and capacity than do larger workplaces (20).

Only 1 strategy had high frequency in both surveys (Food and Drug Administration–approved tobacco cessation medications provided at no or low cost); however, 3 strategies were in the bottom quartile for both instruments: labeling foods with nutrition information, pricing to encourage purchase of healthy options, and use of point-of-decision prompts to encourage physical activity. Comparison across strategies indicated the least variation in lactation support, and greatest variation in nutrition. Both instruments also demonstrated that most users engaged in specific organizational support and culture of wellness and in physical activity-related strategies, such as strong organizational commitment to wellness (eg, support of health promotion at senior management levels) and use of discounts to exercise facilities (eg, off-site gym memberships, onsite repurposed space for exercise classes).

The questions were analyzed by strategy type to look for comprehensiveness across multiple levels of influence. Users of WorkHealthy America consistently implement at least 1 health promoting strategy for each intervention type, ranging from 90% to 99% (Table 3). Additional analysis showed WorkHealthy America employers are more likely to engage in at least 1 strategy across all 5 intervention types compared with ScoreCard users (85% vs 45%, respectively), although the ScoreCard has demonstrated 84% of users engaging in at least 3 different intervention types.

**Table 3:** Percentage of Employers Implementing at Least 1 Health-Promoting Strategy Per Intervention Type, Prevention Partners’ WorkHealthy America (N = 258) and CDC Worksite Health ScoreCard (N = 1,130), September 30, 2013, Through September 30, 2015

Intervention Type	Prevention Partners WorkHealthy America, N (%)	CDC Worksite Health ScoreCard, N (%)
Leadership	248 (96)	788 (70)
Program	256 (99)	773 (69)
Policy	253 (98)	1,045 (93)
Benefits	232 (90)	751 (67)
Environmental supports	245 (95)	1,008 (90)

## Conclusion

Collectively, the ScoreCard and WorkHealthy America have reached 1,797 workplaces across 42 states; these workplaces are diverse in size, sector, and location. More employers (85% of those using WorkHealthy and 45% of those using the ScoreCard) are moving toward a comprehensive approach to worksite wellness by implementing strategies across various levels of influence, including policies, benefits, and environmental supports, that reach all employees and are more sustainable over time. Strategies used by employers to address chronic disease risk factors vary, and future analysis will allow us to better understand this variation and the factors and barriers contributing to the implementation of recommended practices.

This is the first combined analysis of 2 national organization-level healthy workplace data sets. Whereas previous articles have descriptively compared organizational assessments, ours is the first to directly compare data collected by 2 such instruments. The data are not representative; however, they provide practitioners and employers with an inventory of effective healthy workplace strategies and reference points that paint a bigger picture of current practices than either data set alone. The data also fill a critical gap in that no recent, publicly available surveillance data exist on healthy workplace practices. Finally, this analysis is a response to a broader movement in the field to identify and publicly share a set of common measures to benchmark healthy workplaces, and we hope it will advance a national conversation about the importance and need for shared measures across scorecards.
